# Preparation and Optical Properties of Infrared Transparent 3Y-TZP Ceramics

**DOI:** 10.3390/ma10040390

**Published:** 2017-04-07

**Authors:** Chuanfeng Wang, Xiaojian Mao, Ya-Pei Peng, Benxue Jiang, Jintai Fan, Yangyang Xu, Long Zhang, Jingtai Zhao

**Affiliations:** 1 State Key Laboratory of Advanced Special Steel, Shanghai Key Laboratory of Advanced Ferrometallurgy, School of Materials Science and Engineering, Shanghai University, Shanghai 200444, China; wangchuanfeng.052@163.com (C.W.); shuxyy@outlook.com (Y.X.); 2 Key Laboratory of Materials for High Power Laser, Shanghai Institute of Optics and Fine Mechanics, Chinese Academy of Sciences, Shanghai 201800, China; mimi04849@gmail.com (Y.-P.P.); jiangsic@foxmail.com (B.J.); jtfan@siom.ac.cn (J.F.); 3 Key Laboratory of Optoelectronic Devices and Systems of Ministry of Education and Guangdong Province, College of Optoelectronic Engineering, Shenzhen University, Shenzhen 518060, China

**Keywords:** transparent 3Y-TZP ceramics, infrared windows and domes, emittance

## Abstract

In the present study, a tough tetragonal zirconia polycrystalline (Y-TZP) material was developed for use in high-speed infrared windows and domes. The influence of the preparation procedure and the microstructure on the material’s optical properties was evaluated by SEM and FT-IR spectroscopy. It was revealed that a high transmittance up to 77% in the three- to five-micrometer IR region could be obtained when the sample was pre-sintered at 1225 °C and subjected to hot isostatic pressing (HIP) at 1275 °C for two hours. The infrared transmittance and emittance at elevated temperature were also examined. The in-line transmittance remained stable as the temperature increased to 427 °C, with degradation being observed only near the infrared cutoff edge. Additionally, the emittance property of 3Y-TZP ceramic at high temperature was found to be superior to those of sapphire and spinel. Overall, the results indicate that Y-TZP ceramic is a potential candidate for high-speed infrared windows and domes.

## 1. Introduction

Polycrystalline transparent ceramics, such as Al_2_O_3_, MgAl_2_O_4_, MgO, and Y_2_O_3_, are utilized as infrared windows and domes because of their favorable mechanical and optical properties [1]. However, new challenges arise for infrared windows and domes as aircrafts reach supersonic or even hypersonic speeds. One challenge is to protect the window and dome from damage by particle impact. Collisions with raindrops are a problem at the velocities of airplanes and missiles. A simple, empirical correlation is that the damage threshold velocity is proportional to the logarithm of the fracture toughness of the window material. Hence, a material with high strength and high fracture toughness is a potential candidate for use in high-speed infrared windows and domes. In addition, aerothermal heating of such materials when passing through the atmosphere heats the outside surface more rapidly than the inside surface, causing significant stress between the expanded and unexpanded parts. Therefore, high-speed window and dome materials must possess high strength to resist these thermal shock effects. Another problem for hot windows or domes is that the radiation emitted from the window can be so large that it obscures radiation from the object being observed. In this case, an infrared material with a long wavelength cutoff edge is desired, because it results in low emissivity.

In recent years, there has been significant progress in the fabrication of high quality infrared materials [2,3,4,5,6,7,8,9]. Y_2_O_3_–MgO nanocomposite was recently reported to have excellent mid-infrared transmittance over three- to seven-micrometer wavelength ranges with improved mechanical properties over that of pure yttria and magnesia polycrystalline dense ceramics [10,11,12,13]. However, harsher mechanical and thermal environments have imposed more stringent requirements for improved mechanical strength and optical properties of infrared transparent windows and domes.

Yttria-stabilized tetragonal zirconia polycrystalline (Y-TZP) ceramics, commonly containing 3 mol % yttria, have excellent strength, toughness, wear resistance, and chemical resistance compared with cubic zirconia (8% Y_2_O_3_ in addition) [14,15], which is mainly due to the phase transformation toughening effect [16,17]. As a tetragonal material, grain boundary birefringence is an important issue for the transmittance of polycrystalline transparent ceramics [18,19]. More recently, Klimke et al. [20] revealed that if grain sizes are controlled to be much smaller than the wavelength, the influence of birefringent scattering at the grain boundaries can be eliminated. Zhang [21] and Casolco [22] manufactured infrared transparent 3Y-TZP ceramics with sub-micron grain sizes by Spark Plasma Sintering (SPS). The fabricated 3Y-TZP ceramics were found to be potentially useful as high-speed infrared windows and domes [23,24], and their optical properties were amenable to meet the requirements of infrared windows and domes.

Accordingly, the objective of the current study was to fabricate highly infrared transparent Y-TZP ceramics by the hot isostatic pressing (HIP) method. The evolution of the grain size morphology was observed during the sintering process. Additionally, the characteristics of the transparent 3Y-TZP ceramics at both room temperature and high temperature were studied.

## 2. Results and Discussion

**Figure 1:**
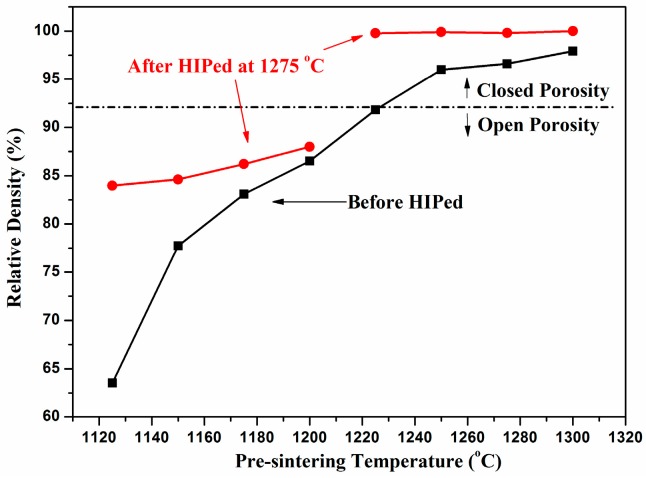
Relative densities of yttria-stabilized tetragonal zirconia polycrystalline (Y-TZP) ceramics containing 3 mol % (3Y-TZP) as a function of pre-sintering temperature (black line and squares), and the corresponding densities after the samples were hot isostatic pressing (HIPed) at 1275 °C (red line and circles).

Figure 1 shows the relative densities of the 3Y-TZP ceramics pre-sintered at different temperatures and their corresponding densities they were after HIPed at 1275 °C. Clearly, the density of the pre-sintering samples increases continuously from approximately 63% to 97% as the sintering temperature increased from 1250 °C to 1300 °C. However, after the samples were subjected to HIP at 1275 °C for two hours, the densities could be divided into two categories. The HIPed samples were essentially fully dense when they were pre-sintered above 1225 °C, whereas the densities of HIPed samples pre-sintered at 1200 °C or lower were approximately 83%–87%, which is much smaller than those pre-sintered at 1225 °C. It is possible that the remaining pores close when the pre-sintering temperature is over 1225 °C, and they could be fully removed during the high-pressure HIP treatment. In contrast, most of the pores are open when the pre-sintering temperature is too low, making them difficult to eliminate since the pores contain many more gas molecules during the HIP treatment.

**Figure 2:**
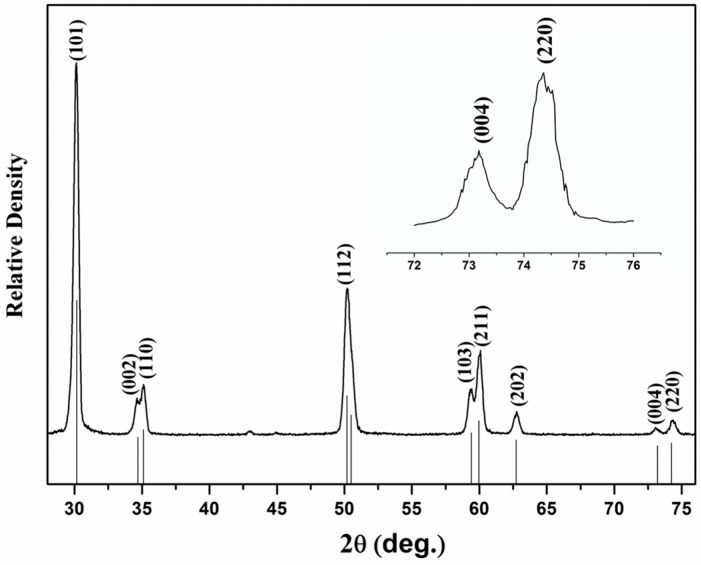
XRD patterns of 3Y-TZP ceramic after HIP treatment.

The X-ray diffraction patterns of the samples HIPed at 1275 °C are consistent with standard tetragonal zirconia (PDF#48-0224), as shown in the Figure 2. For this reason, it could be surmised that pure tetragonal phase was obtained. To distinguish tetragonal reflection peaks from (400)_c_ cubic phases [25], the profiles were enlarged in the 2*θ* range of 72°–76°. Notably, the absence of (400)_c_ between the (004) and (220) reflection peaks excludes the presence of cubic ZrO_2_. The grain size of the 3Y-TZP ceramics were around 200 nm, calculated by using Scherrer’s equation.

**Figure 3:**
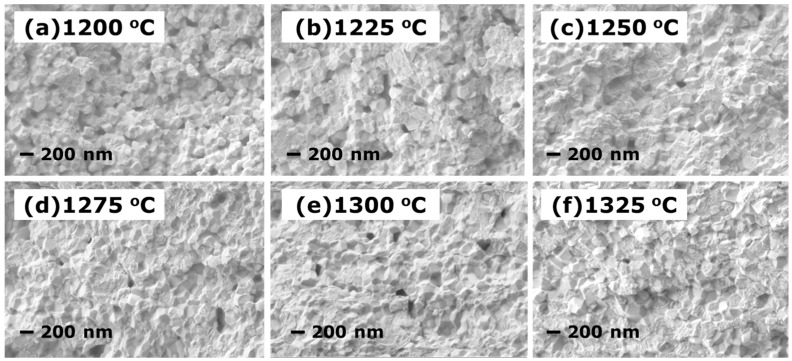
SEM micrographs of the fracture surfaces of compacts pre-sintered at (**a**) 1200 °C; (**b**) 1225 °C; (**c**) 1250 °C; (**d**) 1275 °C; (**e**); 1300 °C and (**f**) 1325 °C.

To determine the microstructural evolution in the densification process, SEM observation was performed on various 3Y-TZP samples after pre-sintering at different temperatures. Figure 3 shows the sample microstructure evolution at pre-sintered temperatures of 1200–1325 °C for two hours. Figure 3a shows the microstructure of the sample sintered at 1200 °C, which contains a large number of connected intergranular pores. As the pre-sintering temperature increased, open pores evolved to closed pores, as shown in Figure 3b–e, with a slightly increased grain size. This tendency of the microstructure is consistent with the change in density indicated in Figure 1. Higher temperatures give rise to a strong densification driving force with rapid grain boundary migration. The densification kinetics was accelerated at 1325 °C, leading to almost fully dense bodies, as shown in Figure 3f.

**Figure 4:**
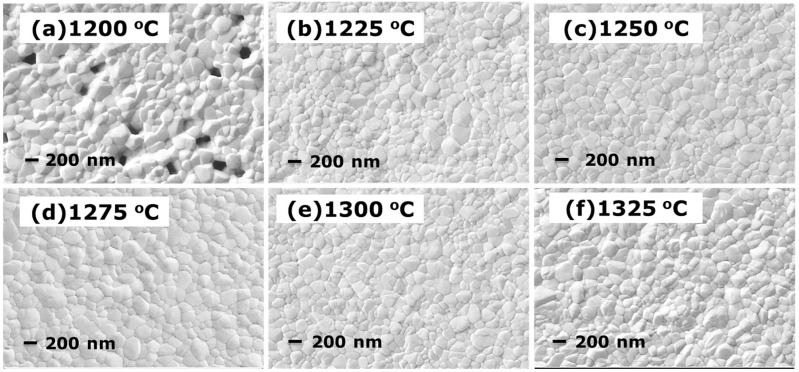
SEM micrographs of samples HIPed at 1275 °C with a pre-sintering temperature of (**a**) 1200 °C; (**b**) 1225 °C; (**c**) 1250 °C; (**d**) 1275 °C; (**e**) 1300 °C and (**f**) 1325 °C.

Figure 4 shows the SEM images of the 3Y-TZP ceramic surfaces after the HIP treatment at 1275 °C. The average grain sizes are approximately 200 nm for all samples pre-sintered at the temperature range between 1200 °C and 1325 °C. Image analysis of the recorded surfaces revealed a variety of intergranular pores trapped in the samples (Figure 4a). The intergranular pores could not be eliminated through the HIP treatment. However, dense 3Y-TZP ceramics were obtained for all samples pre-sintered at 1225–1325 °C after the HIP treatment, as shown in Figure 4b–e, except the sample shown in Figure 4a, which was pre-sintered at 1200 °C. These microstructures confirm that the density changes in the 3Y-TZP ceramics following HIP. This treatment preferentially eliminates closed pores of the pre-sintered samples, which have a relative density over 92% (as indicated in Figure 1), but it has a weak influence on open pores.

**Figure 5:**
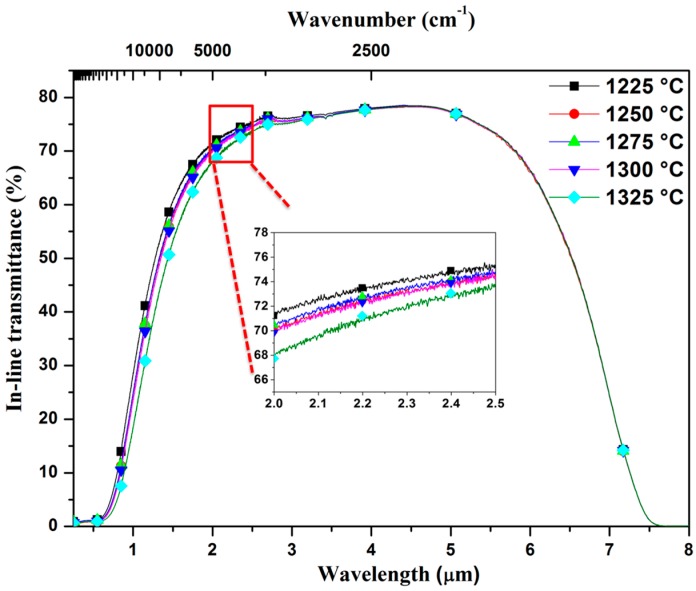
Influence of pre-sintering temperature on the in-line transmittance of 3Y-TZP ceramics HIPed at 1275 °C. Inset: the amplified in-line transmittance micrographs range from 2 μm to 2.5 μm.

The optical in-line transmittance spectra of the samples HIPed at 1275 °C with a thickness of one millimeter are shown in Figure 5. The transmittance curves of all samples have similar slopes and their transmittances increase gradually with wavelengths from three to five micrometers because of their refractive index shift. The sample pre-sintered at 1225 °C followed by HIP at 1275 °C has the highest transmittance of 76.1%–78% in the three- to five-micrometer IR region. Due to the high refractive index of 2.0 [26], the transmittance value is very close to the theoretical transmittance for 3Y-TZP in the IR region. This result is significantly better than that previously reported using spark plasma sintering [21]. Furthermore, according to the lower sintering activity after the samples experienced higher temperatures, it is clear that the in-line transmittance of 3Y-TZP is inversely proportional to the pre-sintering temperature.

**Figure 6:**
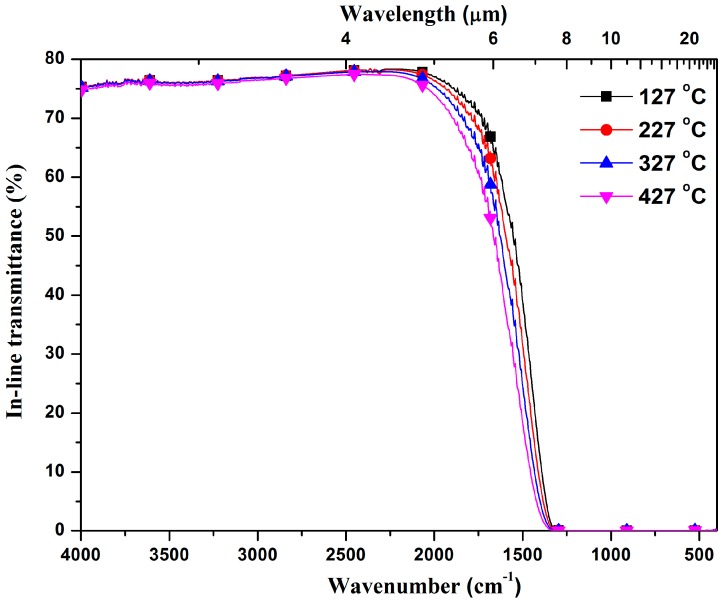
In-line transmittance of 3Y-TZP ceramics in the elevated temperature range from 127 °C to 427 °C.

Figure 6 shows the relationship between the in-line transmittance and elevated temperatures for 3Y-TZP ceramics with a thickness of one-millimeter that were pre-sintered at 1225 °C and HIPed at 1275 °C. Clearly, the in-line transmittance remains stable within a very wide infrared range throughout the temperature range of 127–427 °C. However, there is an obvious degradation at the infrared cutoff edge as the temperature increases, which occurs because of a high phonon concentration. These results suggest the potential application for transparent 3Y-TZP ceramics to function as infrared windows and domes at 427 °C.

**Figure 7:**
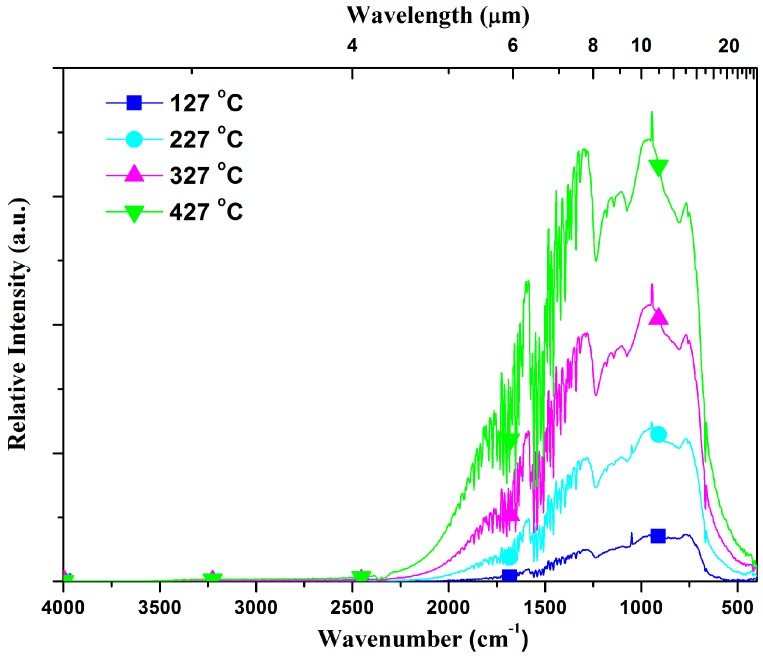
Infrared radiation intensity of a transparent 3Y-TZP sample (25.4 mm diameter, 3.0 mm thick) at high temperature.

According to the measurement process mentioned in Section 2, the spectral relative radiation intensities of transparent 3Y-TZP were measured in the spectral range from 400 cm^−1^ to 4000 cm^−1^ at temperatures ranging from 127 °C to 427 °C. As seen in Figure 7, the radiation power is mainly distributed in the range of 5–15 μm, whereas the radiation intensity is very weak in the mid-infrared region. As expected, the emittance intensity increased significantly as the temperature increased.

**Figure 8:**
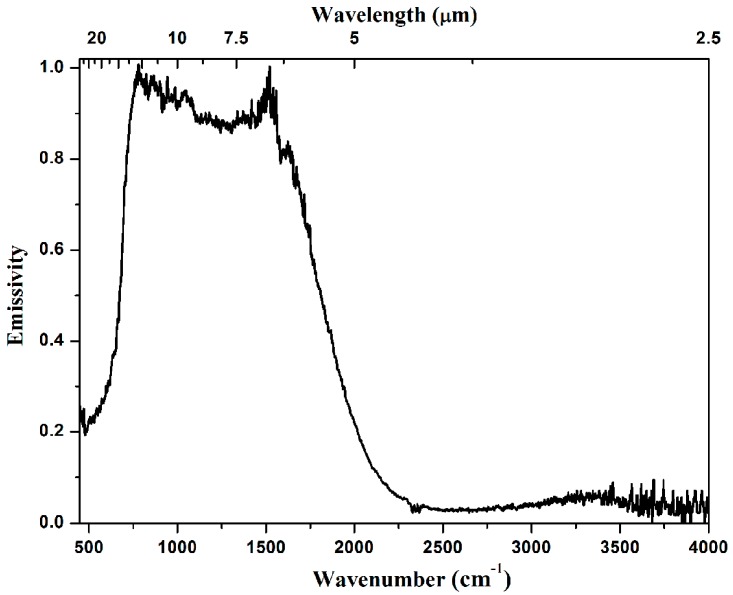
The emittance of a 3Y-TZP sample (25.4 mm diameter, 3.0 mm thick) at 427 °C.

The emissivity of a material is the fraction of the radiation to that emitted by a blackbody. In order to study the emissivity of the prepared ceramics, the emittance of 3Y-TZP at 427 °C, which is the highest temperature investigated in this study, is divided by that of a blackbody at the same temperature. The results are shown in Figure 8. The disturbance at 1600 cm^−1^ and 2350 cm^−1^ are attributed to the background absorption by water and carbon dioxide, respectively [27,28]. As seen, the transparent 3Y-TZP sample has very low emissivity in the mid-infrared range, which is consistent with the high temperature transmittance shown in Figure 6.

**Figure 9:**
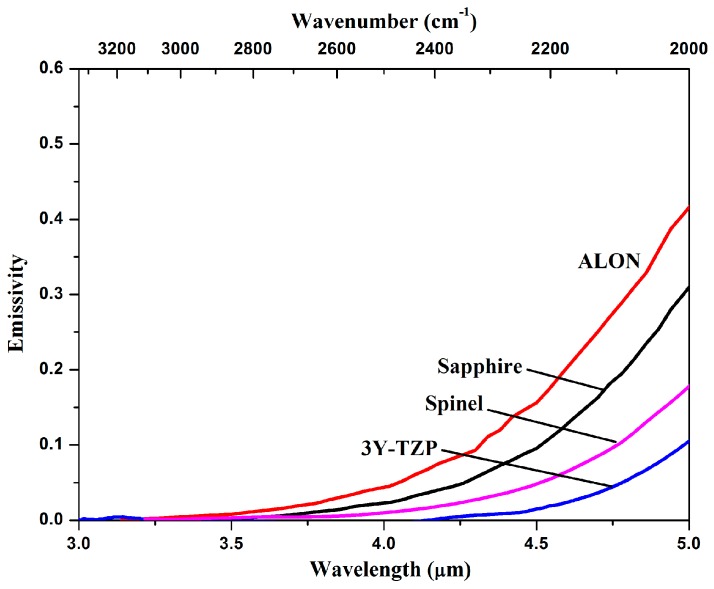
Comparison of the calculated emissivity of ALON, sapphire, spinel (previously reported [29]), and the present 3Y-TZP sample (2.0 mm thick).

The emissivity of common infrared materials calculated by using absorption coefficients have been reported [29]. In order to have a visual understanding of the emissivity of the 3Y-TZP compare with common infrared materials, the emissivity of the 3Y-TZP sample has been normalized to a thickness of two millimeters and plotted in Figure 9, as well as other common infrared materials.

In this comparison, the 3Y-TZP ceramics have the lowest emittance, with a value of only 0.15 at a wavelength of five micrometers under 427 °C, which is mainly due to the longest cutoff wavelength in the absorption spectrum. Clearly, 3Y-TZP ceramics have a distinct emittance advantage over other common infrared materials for wavelengths between three and five micrometers.

**Figure 10:**
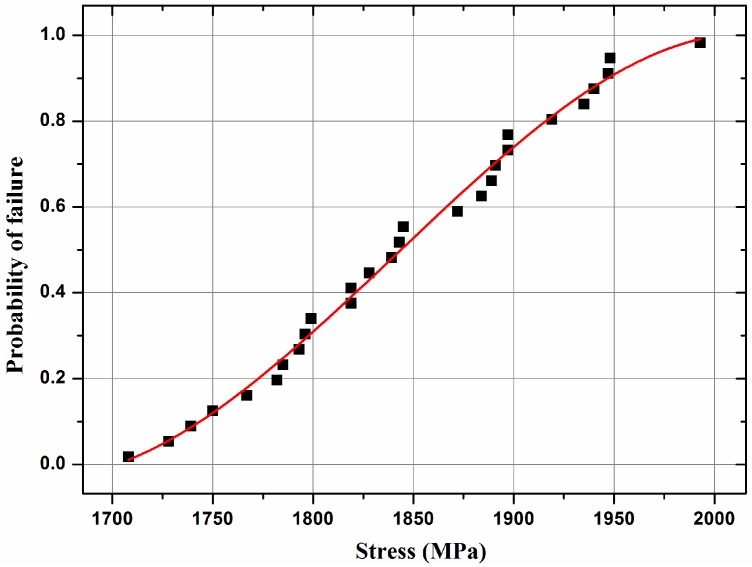
3Y-TZP failure probability as a function of the applied stress.

The mechanical properties of the infrared transparent 3Y-TZP are given in Table 1. As seen, the characteristic bending strength of 3Y-TZP is approximately 1900 MPa and its Vickers hardness is 13.82 GPa. Fracture testing was carried out on a lot of 28 identical disk-shaped (3 × 4 × 36 mm^3^) specimens, Figure 10 displays the Weibull diagram and demonstrates that the recorded strengths obey a two-parameter Weibull distribution remarkably. Weibull parameters *m* = 23.32 and characteristic strength *Sc* = 1888 MPa. The calculation formula and principle refer to reference [11,30,31,32]. The strength is much higher than those of common infrared materials [33,34,35,36]. Further, the measured bending strength values are superior to those measured in previous works of nanocrystalline yttria-stabilized zirconia prepared by hot isostatic pressing [37]. The reason for this could be found in Figure 4, which shows that over stabilization of the tetragonal phase by small grain sizes makes transformation to a monoclinic phase difficult upon the introduction of a crack. According to [38,39,40], the Young’s Modulus, Poisson’s Ratio, expansion coefficient and thermal conductivity of 3Y-TZP are 204 GPa, 0.242, 10.6 × 10^−6^ K^−1^ and 3.2 W·m^−1^·K^−1^, respectively. The thermal shock figure of merit of 3Y-TZP was reach to 2.1 × 10^3^ W/m, calculated by formula reported by Klein [41]:

(1) $$R_{H}\prime =\frac{\sigma_{f}\left(1-\nu \right)}{\alpha E}k$$

where *σ_f_* is mechanical strength, *ν* is Poisson’s ratio, *k* is thermal conductivity, *α* is thermal expansion coefficient, and *E* is Young’s modulus. Thermal shock resistance is favored by high strength, high conductivity, low thermal expansion, and low modulus. Thus, the excellent mechanical properties of the infrared transparent 3Y-TZP ceramics make such materials beneficial for application in harsh environments and aerothermal heating conditions.

## 3. Materials and Methods

The starting material for fabrication was 3 mol % Y_2_O_3_ doped ZrO_2_ powder (Tosoh Corporation, Tokyo, Japan). The powder was uniaxial pressed at 3 MPa and cold isostatic pressed at 210 MPa to prepare green bodies. The resulting green bodies were pre-sintered in air at temperatures ranging from 1200 °C to 1325 °C for 2 h, with heating and cooling rates of 5 °C/min. Subsequently, the pre-sintered samples were treated using HIP under 200 MPa at a temperature of 1275 °C for 2 h in an argon atmosphere. After the samples annealed at 1000 °C for 3 h in air, they were ground and optically polished for characterization.

The densities of the pre-sintered and HIPed bodies were determined using the Archimedes method. Phase identification was performed using XRD with an Empyrean powder diffractometer (PANalytical X’Pert Pro, Eindhoven, The Netherlands). The microstructural evolutions of the polished pre-sintered and HIPed 3Y-TZP samples were studied using field-emission scanning electron microscopy (FE-SEM, Auriga S40, Zeiss, Oberkochen, Germany). The average grain size was determined from scanning electron micrographs of the thermally etched surfaces by the linear analysis intercept technique using a factor of 1.56 times the average intercept length [42]. Transmittance of the polished 3Y-TZP ceramics (thickness of 1 mm) was measured over the wavelength region from 0.25 μm to 8 μm using a Lambda 750 UV/VIS/NIR spectrophotometer (Perkin Elmer, Waltham, CT, USA) and a Nicolet 6700 Fourier-transform infrared (FT-IR) spectrometer (Thermo Nicolet, Youngstown, OH, USA). The three-point bending strength of the samples was measured on 3 × 4 × 36 mm^3^ test bars (10 bars for the test) by a universal testing machine (Instron-5592, Instron Corporation, Norwood, MA, USA). The Vickers hardness and fracture toughness of the samples were measured at room temperature using the indentation method with a load of 98 N for a dwell time of 15 s. The diagonal and crack lengths were measured using optical microscopy, and each data point corresponded to an average number of 6–10 indentations.

**Figure 11:**
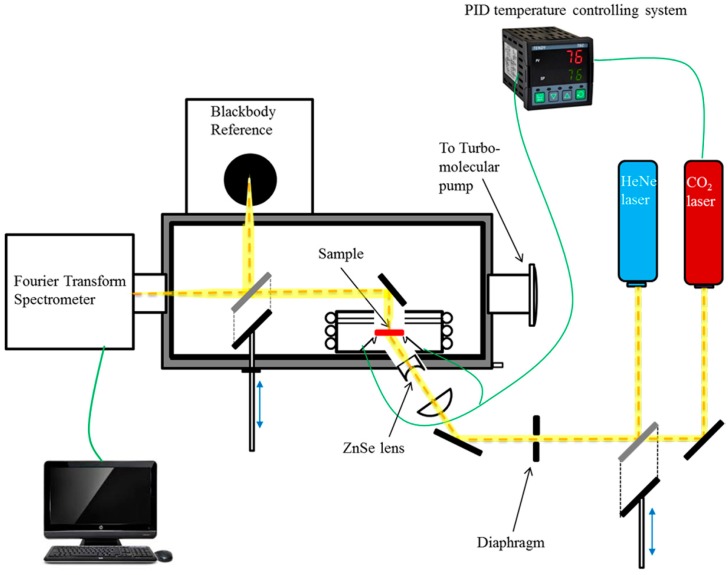
Schematic diagram of the emittance measurement apparatus.

For the high temperature in-line transmittance measurements, the samples were placed in a mini tube furnace located at the center of an FT-IR spectrometer, which can heat the sample to 427 °C. A schematic diagram of the emission measurement system is shown in Figure 11. The measurement apparatus includes a CO_2_ laser to heat the sample, a blackbody reference to calibrate the emittance, and a Fourier transform infrared spectrometer (FT-IR, Thermo Nicolet) to measure the infrared radiation emitted from the samples and blackbody. The spectrometer recorded the real-time radiation of the sample and blackbody at each temperature.

## 4. Conclusions

In the present paper, highly infrared (IR) transparent 3 mol % tetragonal zirconia polycrystalline (3Y-TZP) materials were fabricated by hot isostatic pressing (HIP) sintering. A high transmittance of 76.1%–78% in the three- to five-micrometer IR region was achieved for samples with a grain size of approximately 200 nm. The in-line transmittance remained stable as the temperature increased to 427 °C, with degradation occurring only near the infrared cutoff edge. The emittance of 3Y-TZP at 427 °C was less than those of sapphire and spinel. Overall, this study highlights the great potential of this type of material for hypersonic infrared windows and domes.

## Figures and Tables

**Table 1:** Mechanical properties of 3Y-TZP transparent ceramics.

Materials	Characteristic Bending Strength (Mpa)	Vickers Hardness (Gpa)
3Y-TZP	1888	13.82
